# Exosomes: A Potential Key Target in Cardio-Renal Syndrome

**DOI:** 10.3389/fimmu.2014.00465

**Published:** 2014-10-08

**Authors:** Laura Gonzalez-Calero, Marta Martin-Lorenzo, Gloria Alvarez-Llamas

**Affiliations:** ^1^Immunology Department, Instituto de Investigación Sanitaria Fundación Jiménez Díaz, Universidad Autónoma de Madrid, Madrid, Spain

**Keywords:** exosomes, cardiovascular, kidney, proteomics

## Abstract

Exosomes have proven roles in regulating immune response, antigen presentation, RNA and protein transfer, and cell–cell (organ–organ) interaction/signaling. These microvesicles can be considered a mechanism of non-classical secretion of proteins, and they represent a subproteome, thus assisting in the difficult task of biomarker discovery in a biological fluid as urine, plasma, or serum. A potential role of exosomes in the cardio-renal syndrome is currently underexplored. Cardiovascular disease continues to be the leading cause of morbidity and mortality worldwide and, particularly, rates of cardiovascular events and death consistently increase as kidney function worsens. In other words, chronic kidney disease acts as a risk multiplier. Unfortunately, the relationship between markers of cardiovascular risk in kidney pathology often differs from that in the general population. Efforts in the search for novel action mechanisms simultaneously operating in both pathologies are thus of maximum interest. This article focuses to the role of exosomes in cardiovascular and renal diseases, in the search for novel key targets of interaction between heart and kidneys.

## The Cardio-Renal Syndrome

The term “cardio-renal syndrome” (CRS) encloses a scenario of clinical interactions in which cardiac and renal dysfunction coexists (1). The cross-talk between the heart and the kidneys is clearly evidenced but not fully understood. Observational and clinical data showed that acute/chronic worsening of kidney function directly contributes to acute/chronic cardiac disease and vice versa, constituting the CRS.

Chronic kidney disease (CKD) is increasingly recognized as a major public health problem and its prevalence is continuously rising. Defined as an irreversible progressive loss of renal function for 3 months or longer, it is categorized into five stages of increasing severity (CKD1-5). In early stages, it develops silently and asymptomatic, which enormously difficult early diagnosis and intervention. In CKD patients, rates of cardiovascular events and death consistently increase as kidney function becomes worse. Thus, CKD acts as a risk multiplier. The risk of unfavorable cardiovascular prognosis increases as renal insufficiency progresses toward end stage renal disease (ESRD), reaches its maximum, and persists following successful renal transplantation. Subjects with mild to moderate renal dysfunction have a higher probability of dying by a cardiovascular event than due to the kidney disease itself before reaching ESRD. In fact, the American Heart Association (AHA) stated that CKD patients should be regarded as the highest risk group for subsequent cardiovascular disease (CVD) (2).

It may be thought that risk factors and misregulated molecules known to be acting in CVD in the general population can be directly translated into the renal disease frame. However, the relationship between risk markers and CV events in kidney pathology often differs from that in the general population. One of the main reasons is that traditional CV risk factors, e.g., age, male gender, hypertension, diabetes, dyslipidemia, smoking, overweight, and hyperhomocysteinemia are highly prevalent in CKD. Additionally, contribution of risk factors specifically associated to kidney disease should be added, e.g., anemia, calcium-phosphate disorders, electrolyte imbalances, chronic inflammation, oxidative stress, hypercatabolism, uremic condition, and vitamin D deficiency (3). Some clinical trials focus on known drugs acting to control abovementioned risk factors (blood pressure management, lipid lowering, antiplatelet therapy, etc.). Unfortunately, the results are usually unexpected or not convincing, finding a non-clear response in the adequate direction. Additionally, most of the studies about CVD in CKD are carried out from a *post hoc* subgroup analysis of larger trials, which were not specifically designed to investigate effects on CKD population, so that final conclusions should be postponed until further evidence is found (4). In other cases, cardiovascular trials even excluded CKD patients from enrollment. Thus, despite of research efforts, active cardioprotective treatment is underemployed in renal disease patients, probably due to the uncompleted knowledge of the cardio-renal puzzle.

In this context, documented evidence exists to consistently prove that cardiovascular complications should be considered a key, unsolved and state-of-the art issue in kidney disease, in terms of early on-time diagnosis, accurate prognosis prediction, and in-depth understanding of underlying mechanisms.

## Exosomes Reflect Pathophysiological Changes in the Kidney

The classical pathway of exosome formation involves intraluminal vesicles (ILVs) formation within multivesicular bodies (MVBs) whose membrane fuses with the plasma membrane and release the ILVs, called exosomes once secreted (5). A potential role in the pathophysiology of the kidney has been attributed to exosomes, which may participate as mediators of intercellular communication, activate signaling mechanisms in the target cell, transfer their content in mRNAs, miRNAs and proteins, or just constituting a way of cellular contents disposal (6, 7) (Table 1).

**Table 1:** **Overview of the main roles described for exosomes in cardiovascular and renal diseases**.

Sample	Disease	Main findings	Reference
**BIOMARKERS SOURCE**			
Urine	Aldosteronism	pNCC, marker of aldosteronism	(23)
Urine	AKI and podocyte injury	Transcription factors in urinary exosomes	(11)
		ATF3, marker of early AKI	
		WT-1, marker of early podocyte injury	
Urine	Diabetic nephropathy	WT-1, marker of DN	(20)
Urine	Diabetic nephropathy	VDAC1, AMBP, MLL3 markers of DN	(18)
Urine	Renal I/R injury	Aquaporin-1, novel exosomal marker in renal I/R injury	(22)
Urine	DGF in renal transplantation	Exosomal NGAL correlates with DGF patients	(17)
**INTERCELLULAR COMMUNICATION**			
Kidney cortical collecting duct cells		Functional Aquaporin-2 transfer between kidney cells by exosomes	(13)
Urinary exosome-like vesicles		PKD proteins, shed in urinary membrane particles, which interact with primary cilia	(6)
Model of kidney injury		Exosomes produced by injured epithelial cells activate fibroblasts by delivering TGF-b1 mRNA	(7)
Cardiomyocytes		HIF-1α initiates expression of TNF-α mediated by exosomes in hypoxia	(38)
Vascular smooth muscle cells		VSMC-derived exosomes mediate vascular calcification	(39)
Endothelial cells		HSP70 secretion from endothelial cells is exosome-dependent	(40)
**CARDIOPROTECTION**			
MSCs		After ischemic preconditioning, MSCs secrete exosomes enriched with miR-22	(31)
Rats heart		Remote cardioprotection after ischemic preconditioning is mediated by heart extracellular vesicles	(41)
CPCs from mouse hearts		CPC-exosomes, as a therapeutic vehicle for cardioprotection	(34)

*AKI: acute kidney injury; DN: diabetic nephropathy; DGF: delayed graft function; PKD: polycystic kidney disease; MSCs: mesenchymal stem cells; CPC: cardiac progenitor cells*.

*Representative examples*.

In the kidney, exosomes can be released by cells as podocytes, pass through the renal tubule and they can either be uptaken by recipient epithelial cells of the collecting duct, or influence them through secretion of their content. Exosomes finally appear in urine (8, 9). Thus, these vesicles are direct messengers of what is happening in the kidney, both in acute and chronic damage, carrying molecular markers of renal dysfunction and structural injury (10, 11). More than a way of exocytic cell waste elimination, they should be considered as key molecular targets and a valuable source of potential biomarkers, particularly when kidney tissue is not available or as a non-invasive alternative to biopsy-based diagnosis (12). Aquaporin-2 present in exosomes released from collecting duct cells was shown to be physiological regulated and a close reflection of cellular expression, pointing to a novel mechanism of cell-to-cell communication inside the kidney (13). Another example of their role in intercellular communication is the observation that renal brush border-derived exosomes can induce crystallization in nephrolithiasis (14). In the same line, a still underexplored role in transplantation or protection against acute kidney injury (AKI) or toxics negative effect has been attributed to microvesicles in general and exosomes in particular (15, 16). A recent study shows a high presence of NGAL protein in urinary exosomes. This protein has been described as a good marker of AKI, and the reported correlation of urinary exosomal NGAL levels with delayed graft function after kidney transplantation point to the exosomal fraction as a more sensitive substrate to evaluate early biomarkers of prognosis in this context (17).

Zubiri et al. have recently published a panel of altered proteins composed by MLL3, AMBP, and VDAC1 in the urinary exosomes of patients with diabetic nephropathy (DN) compared to healthy controls (18). DN is the main cause of ESRD, but underlying mechanisms are not fully understood and renal damage observed in biopsied tissue does not always correlate with measurable indicators as microalbuminuria. In the search for novel and earlier indicators, urinary exosomes have been investigated in an animal model of DN finding Xaa-Pro dipeptidase and Major Urinary Protein 1 increased or decreased, respectively (19). Quantitation of damaged podocyte in urine has been proposed as an indicator of renal damage; however, it is not feasible for early detection of DN. Podocyte injury can be evaluated by means of Wilm’s Tumor-1 protein levels, and although it could not be detected in urine from focal segmental glomerulosclerosis (FSGS) patients, Kalani et al. recently showed its predominant presence in urinary exosomes of diabetic patients and increased levels when renal function worsens (20). Markers of renal damage in the context of AKI have also been investigated in urinary exosomes pointing to Fetuin-A and Aquaporin-1 proteins (21, 22). The phosphorylated (active) form of the sodium chloride cotransporter (pNCC) was investigated in aldosteronism and discovered as an indicator of the biological activity of aldosterone and, potentially, as clinical biomarker for primary aldosteronism (23).

Thus, interest of exosomal research in kidney pathology is twofold: as a rich source of novel markers, which promisingly reflect what is happening in progressively or acute damaged kidney tissue, and as molecular messengers between the different parts of the nephron contributing to its optimum functionality by the uptake and release of their content.

## Exosomes and Cardiovascular Disease

Atherosclerosis develops silently and progressively. It is a multifactorial disease that starts with endothelium dysfunction, followed by accumulation of inflammatory cells (macrophages, lymphocytes), lipoproteins, lipids, and fibrous tissue in the wall of large arteries, leading to intima hyperplasia and proliferation of vascular smooth muscle cells (VSMC) within the intima. In advanced lesions, necrosis of macrophages derived foam cells and VSMC results in a lipid-rich core covered by a fibrous cap, which protects the lesions from rupture and consists mainly of collagen and extracellular matrix (ECM) proteins. Plaque rupture and the ensuing thrombosis commonly cause the most acute complications of atherosclerosis such as unstable angina or myocardial infarction (MI) (acute coronary syndrome) or stroke (24). The main problem is that we are facing an asymptomatic development of the pathology in which different cellular types are acting simultaneously.

Only recently, a potential implication of exosomes in CVDs has been raised (25) (Table 1). Similar to what has been described in pancreatic β-cells, exosomes could interact with the ECM via exosomal integrins (26) pointing to a role in plaque unstability or, in the opposite way, in fibrous cap protection. Several studies show an increased amount of microvesicles released in CVD and, in particular, platelets are known to release exosomes, which may be involved in the complex cross-talk among different cell types during atherosclerosis development (27). The capacity of exosomes released by cardiomyocytes to transfer DNA and RNA to different cells has been shown (28). However, the exosomes involvement either in vascular diseases or in cardioprotection mechanisms has not been deeply investigated and fully unraveled. In this sense, preceded by studies showing that cardiac and circulating miRNAs are altered following MI, intercellular communication between heart and bone marrow through released exosomal miRNAs has been hypothesized as a protective/regeneration mechanism after ischemia (29–31). Apparently, similar mechanisms to those operating in preconditioning take place in cardioprotection conferred by exosomes (32–34).

Additionally, exosomal surface can be functionalized to direct them toward a specific target. In this way, they can be converted into therapeutic transporters as they may be acting on a solely cellular type (i.e., cardiac cells). The drug efficiency could be so increased and side effects could be minimized. In a similar way, exosomes have been proposed as ideal therapeutic agents in regenerative medicine, particularly in stem cell based therapies to treat acute MI (35).

## Perspectives: Exosomes, a Novel Target in the Cardio-Renal Puzzle

Deeping insight the cardio-renal puzzle demands for expanded knowledge of many still missing pieces. This challenging multidisciplinary task should be accomplished in two main directions: discovery of novel markers of disease and understanding the underlying physiopathological mechanisms taking place. In general, biomarkers can be classified attending to the kind of information provided in (a) risk assessment, (b) screening markers to distinguish between healthy and pathologic condition, (c) prognostic markers able to predict course of disease or therapy effect, (d) stratification markers to envisage responders and non-responders to drug, and (e) therapy monitoring, able to monitor the efficacy of treatment once the responder status is established (36). The difficulties behind novel biomarkers discovery make sense particularly in relation to approach cardiac or renal tissue.

Totally different from other diseases as cancer pathology in which biopsy procedures are routinely indicated, it is very much difficult, when not unfeasible, directly approach the heart or the kidney to investigate pathological changes of significance. In certain disorders as DN, a diagnosis based on clinical evidences usually takes place late in the course of disease or when damage at tissue level is already irreversible. Atherosclerosis silent and asymptomatic development results in the worst case in a fatal event, and there is lack of effective preventive markers to stratify risk and design preventive measurements individually.

Biological fluids such as serum, plasma, or urine are being explored in the different contexts in the search for diagnostic, prognostic, or risk markers. One of the main difficulties is the wide dynamic range of the sample when proteins are to be investigated. Fishing low-abundance molecules can be facilitated when approaching a certain subproteome such as the cellular or tissue secretome. In the same way, isolation of exosomal fraction from a biological fluid or secretome allows narrowing the complexity of the sample and focusing the search in a more specific target (37). Exosomal isolation from the original source (cell/tissue secretome, plasma, urine, platelets, etc.) is not a trivial task, and it should be carefully approached. Indeed, particular limitations has been pointed to in terms of “purity” of isolated exosomes, in the sense that care should be taken whenever a specific role is to be attributed specifically to these microvesicles instead of to microparticles or apoptotic blebs or mixtures of both. In urine, exosomes represent only a 3% of the whole proteome, thus they constitute an enriched subproteome with reduced complexity compared to the whole urine. In this way, low-abundance molecules that may have pathophysiological significance are enriched against high-abundance ones. Their value as source of potential markers of disease is exemplified when the exosomal content can directly reflect the tissue situation.

Apart from a source of potential novel markers, they act as a way of intercellular communication, which although proved with sufficient evidence is currently underexplored. This exosomal role has been shown, for instance, in terms of proteins and miRNAs transfer from the origin cell and the target cell or acting as functionalized messengers to ensure specific drug delivery to the desired point of action. Related to cardioprotection following an acute cardiovascular event (e.g., MI) or to ameliorate the consequences of ischemia-reperfusion, there are evidences pointing to an active role for exosomes in ischemic signaling, myocardial repair, and communication between heart and bone marrow. This exosomal role acquires special significance in the context of CRS in which the cross-talk between heart and kidneys is known but not gone through.

**Figure 1:**
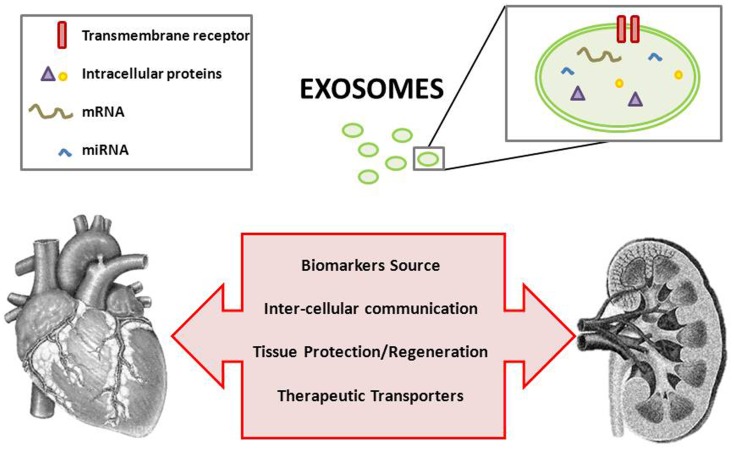
**Exosomes and the cardio-renal puzzle**.

Once overcome methodological difficulties, exosomal research in the context of cardio-renal pathology is therefore more than justified (Figure 1). Exosomes are currently being of increased consideration and they constitute a promising field in this and other pathologies.

## Conflict of Interest Statement

The authors declare that the research was conducted in the absence of any commercial or financial relationships that could be construed as a potential conflict of interest.

## References

[1] Cruz DN Cardiorenal syndrome in critical care: the acute cardiorenal and renocardiac syndromes. Adv Chronic Kidney Dis (2013) 20:56–66 10.1053/j.ackd.2012.10.005 23265597

[2] Sarnak MJ Levey AS Schoolwerth AC Coresh J Culleton B Hamm LL Kidney disease as a risk factor for development of cardiovascular disease: a statement from the American heart association councils on kidney in cardiovascular disease, high blood pressure research, clinical cardiology, and epidemiology and prevention. Hypertension (2003) 108:2154–69 10.1161/01.CIR.0000095676.90936.80 14581387

[3] Stenvinkel P Carrero JJ Axelsson J Lindholm B Heimbürger O Massy Z Emerging biomarkers for evaluating cardiovascular risk in the chronic kidney disease patient: how do new pieces fit into the uremic puzzle? Clin J Am Soc Nephrol (2008) 3:505–21 10.2215/CJN.03670807 18184879 PMC6631093

[4] Jun M Lv J Perkovic V Jardine MJ Managing cardiovascular risk in people with chronic kidney disease: a review of the evidence from randomized controlled trials. Ther Adv Chronic Dis (2011) 2:265–78 10.1177/2040622311401775 23251754 PMC3513885

[5] van der Pol E Boing AN Harrison P Sturk A Nieuwland R Classification, functions, and clinical relevance of extracellular vesicles. Pharmacol Rev (2012) 64:676–705 10.1124/pr.112.005983 22722893

[6] Hogan MC Manganelli L Woollard JR Masyuk AI Masyuk TV Tammachote R Characterization of PKD protein-positive exosome-like vesicles. J Am Soc Nephrol (2009) 20:278–88 10.1681/ASN.2008060564 19158352 PMC2637052

[7] Borges FT Melo SA Özdemir BC Kato N Revuelta I Miller CA TGF-b1–containing exosomes from injured epithelial cells activate fibroblasts to initiate tissue regenerative responses and fibrosis. J Am Soc Nephrol (2013) 24:385–92 10.1681/ASN.2012101031 23274427 PMC3582210

[8] Salih M Zietse R Hoorn EJ Urinary extracellular vesicles and the kidney: biomarkers and beyond. Am J Physiol Renal Physiol (2014) 306:F1251–9 10.1152/ajprenal.00128.2014 24694589

[9] Prunotto M Farina A Lane L Pernin A Schifferli J Hochstrasser DF Proteomic analysis of podocyte exosome-enriched fraction from normal human urine. J Proteomics (2013) 82:193–229 10.1016/j.jprot.2013.01.012 23376485

[10] Miranda KC Bond DT McKee M Skog J Păunescu TG Da Silva N Nucleic acids within urinary exosomes/microvesicles are potential biomarkers for renal disease. Kidney Int (2010) 78:191–9 10.1038/ki.2010.106 20428099 PMC4451567

[11] Zhou H Cheruvanky A Hu X Matsumoto T Hiramatsu N Cho ME Urinary exosomal transcription factors, a new class of biomarkers for renal disease. Kidney Int (2008) 74:613–21 10.1038/ki.2008.206 18509321 PMC2562924

[12] Moon PG Lee JE You S Kim TK Cho JH Kim IS Proteomic analysis of urinary exosomes from patients of early IgA nephropathy and thin basement membrane nephropathy. Proteomics (2011) 11:2459–75 10.1002/pmic.201000443 21595033

[13] Street JM Birkhoff W Menzies RI Webb DJ Bailey MA Dear JW Exosomal transmission of functional aquaporin 2 in kidney cortical collecting duct cells. J Physiol (2011) 589:6119–27 10.1113/jphysiol.2011.220277 22025668 PMC3286690

[14] Anderson HC Mulhall D Garimella R Role of extracellular membrane vesicles in the pathogenesis of various diseases, including cancer, renal diseases, atherosclerosis, and arthritis. Lab Invest (2010) 90:1549–57 10.1038/labinvest.2010.152 20805791

[15] Fleissner F Goerzig Y Haverich A Thum T Microvesicles as novel biomarkers and therapeutic targets in transplantation medicine. Am J Transplant (2012) 12:289–97 10.1111/j.1600-6143.2011.03790.x 22082333

[16] Borges FT Reis LA Schor N Extracellular vesicles: structure, function, and potential clinical uses in renal diseases. Braz J Med Biol Res (2013) 46:824–30 10.1590/1414-431X20132964 24141609 PMC3854311

[17] Alvarez S Suazo C Boltansky A Ursu M Carvajal D Innocenti G Urinary exosomes as a source of kidney dysfunction biomarker in renal transplantation. Transplant Proc (2013) 45:3719–23 10.1016/j.transproceed.2013.08.079 24315007

[18] Zubiri I Posada-Ayala M Sanz-Maroto A Calvo E Martin-Lorenzo M Gonzalez-Calero L Diabetic nephropathy induces changes in the proteome of human urinary exosomes as revealed by label-free comparative analysis. J Proteomics (2014) 96:92–102 10.1016/j.jprot.2013.10.037 24211404

[19] Raimondo F Corbetta S Morosi L Chinello C Gianazza E Castoldi G Urinary exosomes and diabetic nephropathy: a proteomic approach. Mol Biosyst (2013) 9:1139–46 10.1039/c2mb25396h 23344851

[20] Kalani A Mohan A Godbole MM Bhatia E Gupta A Sharma RK Wilm’s tumor-1 protein levels in urinary exosomes from diabetic patients with or without proteinuria. PLoS One (2013) 8:e60177 10.1371/journal.pone.0060177 23544132 PMC3609819

[21] Zhou H Pisitkun T Aponte A Yuen PS Hoffert JD Yasuda H Exosomal Fetuin-A identified by proteomics: a novel urinary biomarker for detecting acute kidney injury. Kidney Int (2006) 70:1847–57 10.1038/sj.ki.5001874 17021608 PMC2277342

[22] Sonoda H Yokota-Ikeda N Oshikawa S Kanno Y Yoshinaga K Uchida K Decreased abundance of urinary exosomal aquaporin-1 in renal ischemia-reperfusion injury. Am J Physiol Renal Physiol (2009) 297:F1006–16 10.1152/ajprenal.00200.2009 19640902

[23] van der Lubbe N Jansen PM Salih M Fenton RA van den Meiracker AH Jan Danser AH The phosphorylated sodium chloride cotransporter in urinary exosomes is superior to prostasin as a marker for aldosteronism. Hypertension (2012) 60:741–8 10.1161/HYPERTENSIONAHA.112.198135 22851731

[24] Alvarez-Llamas G de la Cuesta F Barderas MG Darde V Padial LR Vivanco F Recent advances in aterosclerosis-based proteomics: new biomarkers and a future perspective. Expert Rev Proteomics (2008) 5:679–91 10.1586/14789450.5.5.679 18937558

[25] Cosme J Liu PP Gramolini AO The cardiovascular exosome: current perspectives and potential. Proteomics (2013) 13:1654–9 10.1002/pmic.201200441 23526783

[26] Clayton A Turkes A Dewitt S Steadman R Mason MD Hallett MB Adhesion and signaling by B cell-derived exosomes: the role of integrins. FASEB J (2004) 18:977–9 10.1096/fj.03-1094fje 15059973

[27] Heijnen HF Schiel AE Fijnheer R Geuze HJ Sixma JJ Activated platelets release two types of membrane vesicles: microvesicles by surface shedding and exosomes derived from exocytosis of multivesicular bodies and alpha-granules. Blood (1999) 94:3791–9 10572093

[28] Waldenstrom A Genneback N Hellman U Ronquist G Cardiomyocyte microvesicles contain DNA/RNA and convey biological messages to target cells. PLoS One (2012) 7:e34653 10.1371/journal.pone.0034653 22506041 PMC3323564

[29] Sahoo S Losordo DW Exosomes and cardiac repair after myocardial infarction. Circ Res (2014) 114:333–44 10.1161/CIRCRESAHA.114.300639 24436429

[30] Yellon DM Davidson SM Exosomes: nanoparticles involved in cardioprotection? Circ Res (2014) 114:325–32 10.1161/CIRCRESAHA.113.300636 24436428

[31] Feng Y Huang W Wani M Yu X Ashraf M Ischemic preconditioning potentiates the protective effect of stem cells through secretion of exosomes by targeting Mecp2 via miR-22. PLoS One (2014) 9(2):e88685 10.1371/journal.pone.0088685 24558412 PMC3928277

[32] Lai RC Arslan F Lee MM Sze NS Choo A Chen TS Exosome secreted by MSC reduces myocardial ischemia/reperfusion injury. Stem Cell Res (2010) 4:214–22 10.1016/j.scr.2009.12.003 20138817

[33] Arslan F Lai RC Smeets MB Akeroyd L Choo A Aguor EN Mesenchymal stem cell-derived exosomes increase ATP levels, decrease oxidative stress and activate PI3K/Akt pathway to enhance myocardial viability and prevent adverse remodeling after myocardial ischemia/reperfusion injury. Stem Cell Res (2013) 10:301–12 10.1016/j.scr.2013.01.002 23399448

[34] Chen L Wang Y Pan Y Zhang L Shen C Qin G Cardiac progenitor-derived exosomes protect ischemic myocardium from acute ischemia/reperfusion injury. Biochem Biophys Res Commun (2013) 431:566–71 10.1016/j.bbrc.2013.01.015 23318173 PMC3732190

[35] Lai RC Chen TS Lim SK Mesenchymal stem cell exosome: a novel stem cell-based therapy for cardiovascular disease. Regen Med (2011) 6:481–92 10.2217/rme.11.35 21749206

[36] Finley Austin MJ Babiss L Commentary: where and how could biomarkers be used in 2016. AAPS J (2006) 8:E185–9 10.1208/aapsj080122 16596744 PMC2751438

[37] Zubiri I Vivanco F Alvarez-Llamas G Proteomic analysis of urinary exosomes in cardiovascular and associated kidney diseases by two-dimensional electrophoresis and LC-MS/MS. Methods Mol Biol (2013) 1000:209–20 10.1007/978-1-62703-405-0_16 23585095

[38] Yu X Deng L Wang D Li N Chen X Cheng X Mechanism of TNF-α autocrine effects in hypoxic cardiomyocytes: initiated by hypoxia inducible factor 1α, presented by exosomes. J Mol Cell Cardiol (2012) 53:848–57 10.1016/j.yjmcc.2012.10.002 23085511

[39] Kapustin A Chatrou M Kalra S Drozdov I Soong D Furmanik M Regulated exosome secretion by vascular smooth muscle cells mediates vascular calcification. Heart (2014) 100(Suppl 3):A93–4 10.1136/heartjnl-2014-306118.162

[40] Zhan R Leng X Liu X Wang X Gong J Yan L Heat shock protein 70 is secreted from endothelial cells by a non-classical pathway involvingexosomes. Biochem Biophys Res Commun (2009) 387:229–33 10.1016/j.bbrc.2009.06.095 19555663

[41] Giricz Z Varga ZV Baranyai T Sipos P Pálóczi K Kittel A Cardioprotection by remote ischemic preconditioning of the rat heart is mediated by extracellular vesicles. J Mol Cell Cardiol (2014) 68:75–8 10.1016/j.yjmcc.2014.01.004 24440457

